# The Stability of Retrospective Pre-injury Symptom Ratings Following Pediatric Concussion

**DOI:** 10.3389/fneur.2019.00672

**Published:** 2019-06-27

**Authors:** Elizabeth F. Teel, Roger L. Zemek, Kenneth Tang, Gerard Gioia, Christopher Vaughan, Maegan Sady, Isabelle J. Gagnon, the Pediatric Emergency Research Canada (PERC) Concussion Team, Martin H. Osmond

**Affiliations:** Author Affiliations: Department of Pediatrics, Children's Hospital of Eastern Ontario, University of Ottawa, Ottawa, Ontario, Canada; Children's Hospital of Eastern Ontario Research Institute, Ottawa, Ontario, Canada; Department of Pediatrics, Alberta Children's Hospital, Alberta Children's Hospital Research Institute, University of Calgary, Alberta, Canada; Department of Pediatrics, Hospital Ste. Justine, University of Montreal, Montreal, Quebec, Canada; Department of Pediatrics, Children's Hospital of Western Ontario, Western University, London, Ontario, Canada; Department of Pediatrics, Hospital for Sick Children, Toronto, Ontario, Canada; Department of Pediatrics, Manitoba Children's Hospital, Winnipeg, Manitoba, Canada; Department of Pediatrics, Stollery Children's Hospital, Edmonton, Alberta, Canada; Department of Pediatrics, IWK Health Centre, Halifax, Nova Scotia, Canada; Department of Pediatrics, Children's Hospital of Eastern Ontario, University of Ottawa, Ottawa, Ontario, Canada; ^1^School of Physical and Occupational Therapy, McGill University, Montreal, QC, Canada; ^2^Department of Pediatrics and Emergency Medicine, University of Ottawa, Ottawa, ON, Canada; ^3^Children's Hospital of Eastern Ontario Research Institute, Ottawa, ON, Canada; ^4^Department of Pediatric Neuropsychology, Children's National Medical Center, Washington, DC, United States; ^5^Department of Pediatrics, Montreal Children's Hospital, McGill University Health Centre, Montreal, QC, Canada

**Keywords:** mTBI, baseline, children, psychometric, diagnosis

## Abstract

**Objective:** To determine the stability of children's retrospective ratings of pre-injury levels of symptoms over time following concussion.

**Methods:** Children and adolescents (*n* = 3,063) between the ages of 5–17 diagnosed with a concussion by their treating pediatric emergency department (PED) physician within 48 h of injury completed the Post-Concussion Symptom Inventory (PCSI) at the PED and at 1, 2, 4, 8, and 12-weeks post-injury. At each time point, participants retrospectively recalled their pre-injury levels of post-injury symptoms. The PCSI has three age-appropriate versions for children aged 5–7 (PCSI-SR5), 8–12 (PCSI-SR8), and 13–18 (PCSI-SR13). Total scale, subscales (physical, cognitive, emotional, and sleep), and individual items from the PCSI were analyzed for stability using Gini's mean difference (GMD).

**Results:** The mean GMD for total score was 0.31 (95% CI = 0.28, 0.34) for the PCSI-SR5, 0.19 (95% CI = 0.18, 0.20) for the PCSI-SR8, and 0.17 (95% CI = 0.16, 0.18) for the PCSI-SR13. Subscales ranged from mean GMD 0.18 (physical) to 0.31 (emotional) for the PCSI-SR8 and 0.16 (physical) to 0.31 (fatigue) for the PCSI-SR13. At the item-level, mean GMD ranged from 0.13 to 0.60 on the PCSI-SR5, 0.08 to 0.59 on the PCSI-SR8, and 0.11 to 0.41 on the PCSI-SR13.

**Conclusions:** Children and adolescents recall their retrospective pre-injury symptom ratings with good-to-perfect stability over the first 3-months following their concussion. Although some individual items underperformed, variability was reduced as items were combined at the subscale and full-scale level. There is limited benefit gained from collecting multiple pre-injury symptom queries.

**Clinical Trial Registration:** Clinicaltrials.gov through the US National Institute of Health/National Library of Medicine. (NCT01873287; http://clinicaltrials.gov/ct2/show/NCT01873287).

## Introduction

Concussions are a prevalent injury among children and adolescents throughout North America. In the US, concussions account for one in 220 PED visits, accumulating over 700,000 PED visits annually (1–3). Rates of reporting to the emergency department are even higher in Canada, where concussions are responsible for one in every 70 PED visits (4). Currently, an objective biomarker for concussion diagnosis and subsequent recovery is elusive. Instead, health care providers must rely upon a clinical examination in combination with symptom, balance, and cognitive assessments for concussion diagnosis and management (5, 6). This can be particularly difficult when evaluating children and adolescents, especially elementary-aged children, as current clinical assessments are developed for use with adults and are often not validated or developmentally appropriate for young patients (7).

Concussions can result in a number of somatic, emotional, cognitive, or sleep-related symptoms (8), with headache, imbalance/dizziness, and fatigue the most commonly reported post-concussion symptoms (8, 9). Most clinicians do not have access to a concussion symptom checklist completed by the child pre-injury to serve as a baseline level of normal functioning. One alternative approach is to rely on the injured child to retrospectively report pre-injury levels of their current post-injury symptoms. As concussion management requires serial assessment, and potentially oversight from multiple health care providers (10), it may require children to provide retrospective pre-injury ratings multiple times throughout recovery.

The Post-Concussion Symptom Inventory (PCSI) is a commonly used pediatric symptom checklist that is sensitive to concussion (8). The PCSI has three, developmentally appropriate versions for children aged 5–7, 8–12, and 13–18 years old. Several psychometric properties of the PCSI have been previously reported, including its factor structure, internal consistency, rater concordance, and convergent validity (8, 11, 12). The test-retest reliability of the PCSI has been assessed in healthy (i.e., non-injured) children and interclass correlation coefficients ranged from moderate-to-high (0.65 < ICC < 0.89) (8). However, the presence of concussion may alter the stability of this assessment, particularly when retrospectively recalling pre-injury ratings. Adults with concussion are suspected to engage in a “good-old-days” bias, whereby they idealize retrospective pre-injury levels compared to uninjured controls (13, 14). Recall bias in self-reported symptom outcomes is also highlighted in the scientific literature, with patient age and time since event named as important factors (15, 16). Therefore, it is critical to formally evaluate the stability of retrospective pre-injury ratings throughout pediatric concussion recovery, since pre-injury status can have important implications on clinical decision making following injury.

The primary purpose of this study is to determine the stability of retrospectively provided pre-injury ratings from children and adolescents over the first 3-months following a diagnosed concussion. Due to the relatively short follow-up period (12-weeks), we anticipate the effect of a “good-old-days” or recall bias will be relatively minimal and hypothesize that retrospective pre-injury ratings will be highly stable overall. However, we further hypothesize that individuals who report higher retrospective pre-injury symptom ratings will be less stable than individual who report few or no pre-injury symptoms.

## Materials and Methods

### Participants

Data collection methods have been previously described in detail in other studies (17, 18). Briefly, children presenting to nine PEDs across Canada were eligible if they were between 5 and 17 years old, were diagnosed with a concussion using the definition reported by the 2012 Zurich consensus statement (19), presented to the PED within 48 h of injury, and were proficient in either English or French. Children were excluded if they had: (1) A Glasgow Coma Scale score ≤ 13; (2) Any abnormal findings on standard neuroimaging (if neuroimaging was clinically indicated); (3) Neurosurgical operative intervention, intubation, or intensive care stay, (4) Multisystem injuries with treatment requiring hospital stay; (5) Severe developmental delay resulting in communication difficulties; (6) Intoxication at time of PED presentation; (7) No clear primary mechanism of trauma (e.g., sports-related injury, motor vehicle accident, fall) for the current head injury; or (8) Previously enrolled in the same study. Research assistants were present at each PED from 12:00 to 22:00 h to screen for potential participants, assess eligibility, and explain study procedures to eligible patients. This study was carried out in accordance with the recommendations of the Research Ethics Board of each participating institution with written informed consent from all subjects. Written consent was obtained from all eligible and willing parents as well as children and adolescents capable of consenting on their own behalf in accordance with the Declaration of Helsinki. The protocol was approved by the Research Ethics Board of each participating institution.

### Methods and Outcomes

#### Initial PED Visit

Once enrolled, a research assistant collected data from both the parent and child with an electronic survey in their primary language (English or French) on a portable computer tablet to gather demographic information. Along with demographic data, the parent and the child were then asked to complete the age-appropriate version PCSI prior to leaving the PED. Symptoms on the PCSI were rated once scoring the child's current level of post-injury symptoms and once retrospectively recalling the child's pre-injury levels of those same symptoms. In addition to data collection, each treating physician provided a routine clinical assessment of the child, provided discharge instructions, and gave follow-up information as per the normal standard-of-care procedures of that PED site. Prior to leaving the PED, contact information was obtained for all families.

#### Study Follow-Up

Parents chose to complete follow up measures either online using a web-based platform (REDCap) or over the phone (Email: *n* = 2,776, Phone: *n* = 276, Missing: *n* = 11). Families choosing to complete the follow-up sessions over the phone were called by a research assistant, who read out all survey questions and recorded the participant's answers. For families choosing the web-based option, a link to a secure web-based survey was sent to the parent via email. If the survey was not completed within 48 h of the initial reminder email, phone calls were initiated. A maximum of five phone calls per patient was attempted. Once the patient was contacted, ratings of current symptoms and retrospective report of pre-injury levels were collected at all time points (1, 2, 4, 8, and 12-weeks post-enrollment).

#### Post-concussion Symptom Inventory (PCSI)

The PCSI is a developmentally-appropriate concussion symptom scale that was made for children and adolescents. The PCSI has three age-based versions (PCSI-SR5: Ages 5–7, 13-items, 3-point Guttman Scale; PCSI-SR8: Ages 8–12, 25-items, 3-point Guttman Scale; PCSI-SR13: Ages 13–18, 26-items, 7-point Guttman Scale). As the PCSI uses different scaling based on the age-appropriate version, the PCSI-SR5, and the PCSI-SR8 were transformed by multiplying each item by three to make the scale range equivalent to the PCSI-SR13 which used a 7-pt Guttman scale most commonly found in the literature. The PCSI was chosen because it is one of only two symptom scales with age-appropriate version for younger children, with appropriate psychometric data published in the literature (8, 11, 12). Additionally the PCSI-SR8 and PCSI-SR13 can be evaluated as physical, cognitive, emotional, and sleep subscales (8). The list of symptoms and which subscales they contribute to can be found in Table 1.

**Table 1:** List of symptoms provided on each version of the PCSI.

	**PCSI-SR5 ages 5–7**	**PCSI-SR8 ages 8–12**	**PCSI-SR13 ages 13–18**
**POST-CONCUSSION SYMPTOM INVENTORY**			
Physical	1. Have you had headaches? Has your head hurt? 2. Have you felt sick to your stomach like you were going to throw up? 3. Have you felt dizzy? (like things around you were spinning)	1.Have you had headaches? Has your head hurt? 2. Have you felt sick to your stomach or nauseous? 3. Have you had any balance problems or have you felt like you might fall when you walk/run/stand? 4. Have you felt dizzy? (like things around you were spinning) 5. Have bright lights bothered you more than usual? (like when you were in the sunlight, when you looked at lights, or watched TV) 6. Have loud noised bothered you more than usual? (like when people were talking, when you heard sounds, watched TV, or listened to loud music) 7. Have things looked blurry? 8. Have you felt like you are moving more slowly?	1. Headache 2. Nausea 3. Balance problems 4. Dizziness 5. Sensitivity to light 6. Sensitivity to noise 7. Visual problems 8. Move slower than usual 9. Move in a clumsy manner
Sleep		9. Have you felt more tired than usual? 10. Have you felt more drowsy or sleepy than usual?	10. Fatigue 11. Drowsiness
Emotion	4. Have you felt grumpy or irritable? (like you were in a bad mood)	11. Have you felt grumpy or irritable? (like you werein a bad mood) 12. Have you felt sad? 13. Have you felt nervous or worried?	12. Irritability 13. Sadness 14. Nervousness 15. Feeling more emotional
Cognitive	5. Has it been hard for you to pay attention to what you are doing? (like homework or playing games)	14. Has it been hard to think clearly? 15. Has it been hard for you to pay attention to what you are doing? (like homework or chores, listening)to someone, or playing a game? 16. Has it been hard for you to remember things? (likethings you saw or heard, or places you have gone) 17. Have you felt like you are thinking more slowly?	16. Feeling mentally “foggy” 17. Difficulty concentrating 18. Difficulty remembering 19. Answers questions more slowly than usual 20. Gets confused with directions of tasks
	3pt Guttman Scale: 0 = no, 1 = a little, 2 = a lot		7pt Guttman Scale:0 = no problem, 3 = moderate, 6 = severe

### Statistical Analysis

All analyses were completed in *R* version 3.3.2 (Vienna, Austria) (20). Stability was evaluated by quantifying the mean absolute discrepancy of the retrospective pre-injury ratings for each patient, a quantity also known as the Gini's Mean Difference (GMD), and post-injury symptom ratings were not analyzed in this study. GMD is a descriptive outcome calculated by taking the average of the absolute value for all pair-wise comparisons between pre-injury ratings at all study time points (i.e., pre-injury rating at the PED and 1, 2, 4, 8, and 12-weeks follow-ups). GMD was chosen because no reference time-point must be declared (i.e., no single time-point was assumed to be more accurate than others) and it is free from distributional assumptions (i.e., remain valid under non-normal distributions). With six total time points throughout this study, a maximum of 15 pairwise comparisons were possible for each individual item. To be included in the analysis, individuals were required to complete the retrospective pre-injury ratings on the PCSI at least twice. Individuals who only completed the PCSI at one time point were excluded as GMD could not be calculated. Additional information about the amount of missing data present throughout this study can be found in the Supplementary Material.

A GMD value was first calculated using the rating provided for each individual item listed on the PCSI. At the subscale and full-scale level, the GMD value was calculated by taking the average of all constituent items involved (i.e., if the subscale had three items, the GMD values calculated for those three individual-items were averaged to create a GMD value for the entire subscale). This approach allowed all GMD values reported in this study to range from 0 (perfect stability) to 6 (worst possible stability), providing consistency between all levels of analysis (full scale, subscale, and individual-items) and more appropriate comparisons for situations where all individual items were not completed at all time points. To calculate 95% confidence intervals for the GMD values, estimates were provided by percentile bootstrap based on 100 repetitions. As the number of items differs on each version of the PCSI, we chose to report GMD values separately for the PCSI-SR5, PCSI-SR8, and the PCSI-SR13. There is no literature available to define what level of stability is needed in clinical use, but some level of variability is to be expected. We defined an acceptable level of stability to be good-to-perfect to be a GMD <1, which is 1/6th (16.7%) of the possible value given the PCSI range.

Previous concussion studies have shown that ~40% of individuals report no pre-injury level of symptoms (21). Individuals who consistently report no pre-injury levels of problems across all study time-points would have perfect stability (GMD = 0); a large proportion of individuals with perfect stability may mask clinically important findings. Therefore, we completed a subgroup analysis by removing all individuals who reported no pre-injury symptoms at all six time points studied and re-analyzed stability only with individuals reporting some retrospective pre-injury symptom presence throughout the study (+ Pre-Injury Ratings). Stability results for the entire sample and for the + Pre-Injury Ratings subgroup are presented throughout the study.

## Results

A total of 3,063 children aged 5–17 were recruited through the Predicting and Preventing Postconcussive Problems in Pediatrics (5P) study. Of those 3,063 children, 2,866 (93.6%) completed the retrospective pre-injury PCSI rating during at least two time points and were included into the analysis (Ages 5–7: *N* = 499, Ages 8–12: *N* = 1,203, Ages 13–17: *N* = 1,164). In our sample, 207 (41.5%) participants aged 5–7, 343 (28.5%) participants aged 8–12, and 252 (21.6%) participants aged 13–17 retrospectively rated all pre-injury levels as zero throughout the entire study period (GMD = 0) and were not included in the subgroup analysis. Descriptive statistics for the study sample can be found in Table 2.

**Table 2:** Descriptive statistics for the 5–7, 8–12, and 13–18-year-old age groups.

**Item**	**Ages 5–7**				**Ages 8–12**				**Ages 13–18**			
	**No missing data** **(*n* = 306)**	**Some missing data** **(*n* = 193)**	**GMD unavailable** **(*n* = 35)**	***P***	**No missing data** **(*n* = 631)**	**Some missing data** **(*n* = 572)**	**GMD unavailable** **(*n* = 79)**	***P***	**No missing data** **(*n* = 563)**	**Some missing data** **(*n* = 601)**	**GMD Unavailable** **(*n* = 83)**	***P***
Age, median (IQR)	6.7 (5.9, 7.3)	6.6 (5.9, 7.2)	6.6 (5.8, 7.2)	0.30	10.8 (9.7, 11.9)	10.7 (9.5, 11.9)	10.6 (9.5, 11.9)	0.95	15 (13.9, 16.0)	15.2 (14.1, 16.2)	15.2 (14.3, 16.5)	**0.02**
Sex, freq (%)												
Male	184 (60.1)	126 (65.3)	21 (60.0)	0.50	420 (66.7)	368 (64.3)	50 (63.3)	0.67	314 (55.8)	321 (53.5)	53 (63.9)	0.19
Female	122 (39.9)	67 (34.7)	14 (40.0)		211 (33.4)	204 (35.7)	29 (36.7)		249 (44.2)	279 (46.5)	30 (36.1)	
Concussion Hx, freq (%)												
Yes	273 (89.8)	173 (90.1)	30 (93.8)	0.79	528 (83.9)	464 (81.5)	60 (80.0)	0.66	395 (70.7)	374 (62.6)	51 (63.7)	0.16
No	31 (10.2)	17 (9.9)	2 (6.2)		101 (16.1)	105 (18.5)	15 (20.0)		164 (29.3)	223 (37.4)	29 (36.3)	
Personal Hx, freq (%)												
Migraine	16 (5.3)	10 (5.2)	0 (0.0)	0.41	64 (10.2)	60 (10.5)	7 (9.5)	0.96	99 (17.7)	119 (19.8)	17 (21.2)	0.58
Learning disability	8 (2.6)	13 (6.7)	1 (3.2)	0.08	51 (8.1)	39 (6.9)	13 (17.6)	**0.01**	46 (8.2)	62 (10.2)	11 (14.1)	0.19
Attention deficit	12 (3.9)	14 (7.3)	0 (0.0)	0.1	52 (8.3)	53 (9.3)	8 (11.0)	0.67	45 (8.0)	70 (11.7)	14 (17.7)	**0.01**
Anxiety	12 (3.9)	1 (0.5)	0 (0.0)	**0.04**	43 (6.8)	35 (6.1)	3 (4.1)	0.62	65 (11.6)	68 (11.4)	10 (12.7)	0.94
Depression	0 (0.0)	0 (0.0)	0 (0.0)	1	5 (0.8)	7 (1.2)	2 (2.7)	0.3	30 (5.3)	35 (5.8)	8 (10.0)	0.25
Sleep disorder	2 (0.7)	3 (1.6)	0 (0.0)	0.5	8 (1.3)	8 (1.4)	0 (0.0)	0.6	20 (3.6)	17 (2.8)	4 (5.1)	0.53
Other Psychiatric	2 (0.7)	1 (0.5)	0 (0.0)	0.89	5 (0.8)	4 (0.7)	0 (0.0)	0.74	10 (1.8)	8 (1.3)	2 (2.6)	0.65
Mechanism, freq (%)												
Sports/Rec	146 (47.7)	85 (44.3)	10 (31.2)	0.60	436 (69.2)	381 (66.6)	47 (64.4)	0.35	442 (78.5)	470 (78.2)	58 (72.5)	0.07
Non-Sport/fall	155 (50.7)	104 (54.2)	21 (65.6)		179 (28.4)	179 (31.3)	25 (34.2)		91 (16.2)	105 (17.5)	12 (15.0)	
MVC	3 (1.0)	2 (1.0)	1 (3.1)		10 (1.6)	3 (0.5)	0 (0.0)		18 (3.2)	16 (2.7)	4 (5.0)	
Assault	2 (0.7)	1 (0.5)	0 (0.0)		5 (0.8)	7 (1.2)	1 (1.4)		11 (2.0)	10 (1.8)	6 (7.5)	
Other	0 (0.0)	0 (0.0)	0 (0.0)		0 (0.0)	2 (0.3)	0 (0.0)		1 (0.2)	0 (0.0)	0 (0.0)	

*Groups were further evaluated by missing data points, where No Missing Data = participants who completed all time points involved in the study (n = 6), Some Missing Data = participants completed between 2 and 5 time points, and GMD Unavailable = participant only completed one time point (GMD could not be calculated and were not analyzed in this study). Bold values are statistical significance (P < 0.05)*.

### PCSI-SR5

#### Full Sample

The full scale for the PCSI-SR5 was highly stable, with 94% of children displaying good-to-perfect stability. The mean GMD for the full 5–7-year-old sample ranged from 0.13 to 0.60 at the item-level. Dizziness was the most stable individual item and difficulty concentrating showed the lowest stability.

#### + Pre-injury Ratings

When looking only at individuals who retrospectively reported at least one non-zero pre-injury rating throughout the study period, the overall stability was reduced but trends were similar to the full sample. The full scale remained highly stable for the + Pre-Injury sample, with 89% of children continuing to show good-to-perfect. Balance problems and difficulty concentrating remained the most and least stable individual items, respectively. Overall results for the PCSI-SR5 can be found in Table 3.

**Table 3:** Mean (95% CI), median (95% CI), and percentage of individuals with good-to-perfect stability for the PCSI-SR5 (Ages 5–7).

**Level**	**Outcome**	**Stability for PCSI-SR5 (*****n*** **=** **499)**					
		**Mean (95% CI)**		**Median (95% CI)**		**Good-to-perfect stability (N, %)**	
		**Full sample**	**+ Pre-injury ratings only**	**Full sample**	**+ Pre-injury ratings only**	**Full sample**	**+ Pre-injury ratings only**
Item	Headache	0.39 (0.34, 0.46)	1.41 (1.32, 1.52)	0.00 (0.00, 0.00)	1.20 (1.20, 1.50)	413/499 (83%)	54/140 (39%)
	Nausea	0.27 (0.22, 0.33)	1.48 (1.34, 1.64)	0.00 (0.00, 0.00)	1.20 (1.00, 1.60)	447/499 (90%)	39/91 (43%)
	Dizziness	0.13 (0.10, 0.18)	1.52 (1.33, 1.73)	0.00 (0.00, 0.00)	1.35 (1.00, 1.60)	472/499 (95%)	17/44 (39%)
	Irritability	0.42 (0.37, 0.48)	1.52 (1.43, 1.68)	0.00 (0.00, 0.00)	1.50 (1.20, 1.60)	411/499 (82%)	51/139 (37%)
	Difficulty Concentrating	0.60 (0.53, 0.67)	1.60 (1.47, 1.73)	0.00 (0.00, 0.00)	1.50 (1.20, 1.60)	371/499 (74%)	60/188 (32%)
Full Scale	Overall Total	0.31 (0.28, 0.34)	0.53 (0.48, 0.58)	0.20 (0.20, 0.24)	0.40 (0.36, 0.48)	468/499 (94%)	259/290 (89%)

*Outcomes are provided at the item and full-scale level for the full sample and for the + Pre-Injury Ratings subgroup only. No subscales exist in the PCSI-SR5*.

### PCSI-SR8

#### Full Sample

Overall, the full scale for the PCSI-SR8 was highly stable (GMD = 0.19, 95% CI: 0.18, 0.20). The physical subscale showed the highest relative stability, with moving slowly and vision problems encompassing the most stable individual items. The emotional subscale showed the lowest stability among the four subscales. Headache had the lowest relative stability at the item-level, followed by difficulty concentrating.

#### + Pre-injury Ratings

The PCSI-SR8 remained highly stable for the + Pre-Injury sample, but different trends emerged in this group. The physical domain continued to be the most stable subscale, but nausea and headache had the highest relative stability of individual-items. In disagreement with the full sample, fatigue (subscale), and vision problems (item-level) were the least stable relative to other outcomes. Full results for the PCSI-SR8 can be found in Table 4.

**Table 4:** Mean (95% CI), median (95% CI), and percentage of individuals with good-to-perfect stability for the PCSI-SR8 (Ages 8–12).

**Level**	**Outcome**	**Stability for PCSI-SR8 (*****n*** **=** **1,203)**					
		**Mean (95% CI)**		**Median (95% CI)**		**Good-to-perfect stability (N, %)**	
		**Full sample**	**+ Pre-injury ratings only**	**Full sample**	**+ Pre-injury ratings only**	**Full sample**	**+ Pre-injury ratings only**
Item	Headache	0.59 (0.55, 0.64)	1.50 (1.45, 1.55)	0.00 (0.00, 0.00)	1.50 (1.50, 1.60)	904/1,203 (75%)	178/477 (37%)
	Nausea	0.26 (0.22, 0.28)	1.39 (1.32, 1.47)	0.00 (0.00, 0.00)	1.20 (1.09, 1.50)	1,080/1,203 (90%)	99/222 (45%)
	Balance	0.13 (0.10, 0.16)	1.53 (1.36, 1.65)	0.00 (0.00, 0.00)	1.20 (1.00, 1.60)	1,146/1,203 (95%)	43/100 (43%)
	Dizziness	0.14 (0.12, 0.17)	1.43 (1.32, 1.54)	0.00 (0.00, 0.00)	1.20 (1.20, 1.50)	1,133/1,203 (94%)	49/119 (41%)
	Fatigue	0.28 (0.25, 0.31)	1.50 (1.42, 1.59)	0.00 (0.00, 0.00)	1.20 (1.20, 1.50)	1,077/1,203 (90%)	101/227 (45%)
	Drowsy	0.19 (0.17, 0.23)	1.50 (1.40, 1.59)	0.00 (0.00, 0.00)	1.20 (1.20, 1.50)	1,113/1,203 (93%)	65/155 (42%)
	Sensitivity to Light	0.19 (0.17, 0.22)	1.51 (1.42, 1.63)	0.00 (0.00, 0.00)	1.50 (1.20, 1.60)	1,110/1,203 (92%)	62/155 (40%)
	Sensitivity to Noise	0.23 (0.20, 0.26)	1.47 (1.35, 1.56)	0.00 (0.00, 0.00)	1.20 (1.20, 1.55)	1,092/1,203 (91%)	81/192 (42%)
	Irritability	0.36 (0.33, 0.40)	1.43 (1.38, 1.48)	0.00 (0.00, 0.00)	1.35 (1.20, 1.50)	1,029/1,203 (86%)	130/304 (43%)
	Sad	0.25 (0.22, 0.28)	1.45 (1.35, 1.54)	0.00 (0.00, 0.00)	1.50 (1.20, 1.50)	1,080/1,203 (90%)	84/207 (41%)
	Nervous	0.40 (0.36, 0.44)	1.51 (1.43, 1.58)	0.00 (0.00, 0.00)	1.50 (1.20, 1.60)	1,004/1,203 (84%)	118/317 (37%)
	Move Slowly	0.08 (0.07, 0.10)	1.40 (1.30, 1.56)	0.00 (0.00, 0.00)	1.20 (1.00, 1.50)	1,163/1,203 (97%)	32/72 (44%)
	Think Slowly	0.12 (0.09, 0.14)	1.54 (1.43, 1.65)	0.00 (0.00, 0.00)	1.50 (1.20, 1.55)	1,143/1,202 (95%)	35/94 (37%)
	Mental Fog	0.15 (0.13, 0.18)	1.49 (1.40, 1.61)	0.00 (0.00, 0.00)	1.20 (1.00, 1.50)	1,133/1,203 (94%)	52/122 (43%)
	Difficulty Concentrating	0.46 (0.43, 0.51)	1.51 (1.42, 1.57)	0.00 (0.00, 0.00)	1.50 (1.20, 1.60)	978/1,203 (81%)	145/370 (39%)
	Difficulty Remembering	0.25 (0.21, 0.28)	1.47 (1.39, 1.55)	0.00 (0.00, 0.00)	1.50 (1.20, 1.60)	1,080/1,203 (90%)	78/201 (39%)
	Vision	0.10 (0.08, 0.12)	1.57 (1.40, 1.81)	0.00 (0.00, 0.00)	1.20 (1.20, 1.60)	1,158/1,202 (97%)	30/74 (41%)
Subscale	Physical	0.18 (0.17, 0.20)	0.34 (0.32, 0.36)	0.12 (0.12, 0.12)	0.25 (0.23, 0.25)	1,186/1,203 (99%)	632/649 (97%)
	Fatigue	0.23 (0.21, 0.26)	1.08 (1.02, 1.14)	0.00 (0.00, 0.00)	1.00 (0.82, 1.00)	1,104/1,203 (92%)	160/259 (62%)
	Emotional	0.31 (0.29, 0.34)	0.76 (0.73, 0.80)	0.00 (0.00, 0.00)	0.60 (0.60, 0.67)	1,098/1,203 (91%)	390/495 (79%)
	Cognitive	0.22 (0.21, 0.25)	0.60 (0.56, 0.64)	0.00 (0.00, 0.00)	0.45 (0.45, 0.50)	1,140/1,202 (95%)	386/448 (86%)
Full Scale	Overall Total	0.19 (0.18, 0.20)	0.26 (0.25, 0.28)	0.11 (0.09, 0.12)	0.18 (0.18, 0.21)	1,185/1,203 (99%)	841/859 (98%)

*Outcomes are provided at the item, subscale, and full-scale level for the full sample and for the + Pre-Injury Ratings subgroup only*.

### PCSI-SR13

#### Full Sample

The full scale for the PCSI-SR13 had high overall stability. Vision problems, answer slowly, and nausea were the most stable individual items and the physical domain was the most stable subscale. Conversely, fatigue, headache, and the fatigue subscale had the lowest relative stability.

#### + Pre-injury Ratings

The overall stability for the full scale remained high for the + Pre-Injury sample. The physical subscale and dizziness (item) had the highest relative stability. On the low end, fatigue (subscale) and headache (item) had the lowest stability. Overall results for the PCSI-SR13 can be found in Table 5.

**Table 5:** Mean (95% CI), median (95% CI), and percentage of individuals with good-to-perfect stability for the PCSI-SR13 (Ages 13–18).

**Level**	**Outcome**	**Stability for PCSI-SR13 (*****n*** **=** **1,164)**					
		**Mean (95% CI)**		**Median (95% CI)**		**Good-to-perfect stability (N, %)**	
		**Full sample**	**+ Pre-injury ratings only**	**Full sample**	**+ Pre-injury ratings only**	**Full Sample**	**+ Pre-injury ratings only**
Item	Headache	0.39 (0.35, 0.43)	0.97 (0.89, 1.03)	0.00 (0.00, 0.00)	0.80 (0.67, 0.87)	1,015/1,164 (87%)	319/468 (68%)
	Nausea	0.13 (0.11, 0.15)	0.83 (0.76, 0.91)	0.00 (0.00, 0.00)	0.67 (0.60, 0.67)	1,115/1,164 (96%)	131/180 (73%)
	Balance	0.18 (0.15, 0.21)	0.88 (0.81, 0.95)	0.00 (0.00, 0.00)	0.67 (0.60, 0.70)	1,100/1,164 (95%)	178/242 (74%)
	Dizziness	0.15 (0.13, 0.17)	0.78 (0.71, 0.85)	0.00 (0.00, 0.00)	0.60 (0.53, 0.67)	1,117/1,164 (96%)	177/224 (79%)
	Fatigue	0.41 (0.37, 0.44)	0.91 (0.86, 0.97)	0.00 (0.00, 0.00)	0.67 (0.67, 0.87)	1,011/1,164 (87%)	369/522 (71%)
	Drowsy	0.25 (0.21, 0.27)	0.86 (0.80, 0.92)	0.00 (0.00, 0.00)	0.67 (0.60, 0.67)	1,082/1,164 (93%)	252/334 (75%)
	Sensitivity to Light	0.17 (0.14, 0.19)	0.86 (0.78, 0.95)	0.00 (0.00, 0.00)	0.67 (0.60, 0.67)	1,106/1,164 (95%)	166/224 (74%)
	Sensitivity to Noise	0.18 (0.15, 0.20)	0.88 (0.80, 0.96)	0.00 (0.00, 0.00)	0.67 (0.60, 0.80)	1,100/1,163 (95%)	175/238 (74%)
	Irritability	0.38 (0.34, 0.41)	0.93 (0.87, 0.99)	0.00 (0.00, 0.00)	0.73 (0.67, 0.87)	1,006/1,164 (86%)	316/474 (67%)
	Sad	0.23 (0.20, 0.26)	0.93 (0.85, 1.00)	0.00 (0.00, 0.00)	0.67 (0.60, 0.77)	1,082/1,164 (93%)	203/285 (71%)
	Nervous	0.36 (0.32, 0.40)	0.95 (0.89, 1.02)	0.00 (0.00, 0.00)	0.67 (0.67, 0.87)	1,029/1,164 (88%)	305/440 (69%)
	Emotional	0.28 (0.24, 0.31)	0.93 (0.85, 1.01)	0.00 (0.00, 0.00)	0.67 (0.67, 0.80)	1,054/1,164 (91%)	235/345 (68%)
	Move Slowly	0.15 (0.13, 0.17)	0.88 (0.80, 0.96)	0.00 (0.00, 0.00)	0.67 (0.60, 0.77)	1,109/1,164 (95%)	145/200 (73%)
	Mental Fog	0.14 (0.12, 0.17)	0.88 (0.78, 0.95)	0.00 (0.00, 0.00)	0.67 (0.60, 0.80)	1,108/1,164 (95%)	136/192 (71%)
	Concentration	0.36 (0.32, 0.39)	0.92 (0.85, 0.98)	0.00 (0.00, 0.00)	0.67 (0.67, 0.80)	1,042/1,164 (90%)	330/452 (73%)
	Memory	0.25 (0.22, 0.27)	0.87 (0.81, 0.93)	0.00 (0.00, 0.00)	0.67 (0.60, 0.80)	1,082/1,164 (93%)	247/329 (75%)
	Vision	0.11 (0.10, 0.13)	0.84 (0.74, 0.94)	0.00 (0.00, 0.00)	0.67 (0.57, 0.80)	1,128/1,164 (99%)	123/159 (77%)
	Confused	0.22 (0.20, 0.25)	0.86 (0.80, 0.93)	0.00 (0.00, 0.00)	0.67 (0.60, 0.82)	1,086/1,164 (93%)	219/297 (74%)
	Clumsy	0.26 (0.23, 0.29)	0.91 (0.82, 0.98)	0.00 (0.00, 0.00)	0.80 (0.67, 0.87)	1,066/1,164 (92%)	241/339 (71%)
	Answer Slowly	0.13 (0.11, 0.15)	0.81 (0.74, 0.89)	0.00 (0.00, 0.00)	0.60 (0.53, 0.67)	1,127/1,164 (97%)	156/193 (81%)
Subscale	Physical	0.16 (0.14, 0.17)	0.27 (0.24, 0.29)	0.07 (0.04, 0.08)	0.18 (0.15, 0.18)	1,147/1,164 (99%)	671/688 (98%)
	Fatigue	0.31 (0.28, 0.33)	0.65 (0.62, 0.70)	0.00 (0.00, 0.00)	0.50 (0.50, 0.58)	1,081/1,164 (93%)	469/552 (85%)
	Emotional	0.27 (0.25, 0.29)	0.49 (0.45, 0.53)	0.08 (0.08, 0.10)	0.35 (0.30, 0.39)	1,097/1,164 (94%)	572/639 (90%)
	Cognitive	0.18 (0.16, 0.19)	0.36 (0.34, 0.39)	0.00 (0.00, 0.00)	0.24 (0.22, 0.28)	1,133/1,164 (97%)	528/559 (95%)
Full Scale	Overall Total	0.17 (0.16, 0.18)	0.22 (0.20, 0.24)	0.09 (0.08, 0.10)	0.13 (0.12, 0.14)	1,145/1,164 (98%)	892/911 (98%)

*Outcomes are provided at the item, subscale, and full-scale level for the full sample and for the + Pre-Injury Ratings subgroup only*.

## Discussion

The results of this study confirm our hypothesis that the retrospective recall of pre-injury ratings is stable in children and adolescents. At the full-scale level, all mean GMD values were <5% of the scale range, suggesting little dispersion, and high stability in the retrospective recall of pre-injury levels in the 3-months post-concussion. Averaging over all items, including those with very low mean GMDs, and non-consistent retrospective reporting in opposing direction (i.e., retrospectively rating one symptom 1-point higher than previous reports while rating a different symptom 1-point lower than previous reports) likely contribute to the high stability seen across both the full scale and sub-scale level. The wider 95% confidence intervals and smaller percentage of individuals displaying good-to-perfect stability indicate that the youngest children (ages 5–7) may have slightly less stability in their retrospective symptom recall, in agreement with previous literature suggesting young children have the least reliable recall (15). Our results support the high overall stability of retrospective pre-injury ratings despite large, non-linear changes in post-injury symptoms ratings, which have been shown previously in this sample of concussed children and adolescents (22).

Every individual item on each version of the PCSI had a mean GMD ≤ 1/10th of the scale range and a median GMD of 0, indicating little dispersion. Headache, fatigue, and difficulty concentrating were among the least stable individual items across all versions of the PCSI. These particular symptoms are not specific to concussion and are among the most commonly reported baseline symptoms in healthy children and adolescents for their respective age groups (8, 23). Additionally, the severity at which these problems occur if present often fluctuates over time, which can account for the higher variability (less stability) seen in these items. This rationale is supported by previous research in children with headache history, which shows that children can accurately recall headache frequency but struggle with recalling headache intensity and duration (16). Conversely, the items with the highest individual stability (vision, balance/dizziness, and move/answer slowly) are among the least frequently endorsed baseline symptoms in our study, which agrees with previous literature (8).

The PCSI was highly stable as hypothesized. Previous studies show that the “good-old-days” and recall biases can elevate pre-injury symptom reports, which would theoretically decrease the stability of the assessment. However, reports of these biases are traditionally found over longer periods of time than evaluated in this study and in individuals with on-going symptoms (13, 15). The main objective of this study was to describe the overall stability of retrospective symptoms rating on the PCSI. Therefore, stability was examined only as a collective whole over the follow-up period (i.e., not specific to time intervals within the follow-up period) and with pre-injury symptom ratings [post-injury symptom scores and their progression over the follow-up period in this sample have been previously reported (22)]. As such, our methodology does not allow for a conclusive determine regarding the presence of the “good-old-days” in our sample, which should be evaluated further in future studies.

The primary strength of this study lies in the large, diverse sample. Our pediatric participants comprised a wide age range (5–17), included various mechanisms of injury, and embraced patients with behavioral, learning, and psychological problems [more specific information about our sample can be found in Zemek et al. (18)]. Our broad inclusion criteria increase the generalizability of findings. The use of a validated concussion symptom checklist, which has three, developmentally appropriate versions for children and adolescents, is an additional strength. Lastly, the analytical method of GMD is considered a strength due its ability to tolerate non-normally distributed data and handle missing data (only two retrospectively PCSI rating were needed for inclusion). Even though missing data may mean different time intervals between assessments, there is no natural bias toward stability when this is the case which allows the inclusion of more patients and increases generalizability of our results.

Although the psychometric properties of the PCSI are a strength, the number of items and rating scales for the three different versions are not equivalent and represent a limitation. All reported pre-injury rating responses (0–2 scale) on the PCSI-SR5 and PCSI-SR8 were multiplied by three to make all response outcomes range of GMD 0–6. However, this induces more instability into the PCSI-SR5 and PCSI-SR8 and assumes a 1-point discrepancy on the 0–2 scale is equal to a 3-point discrepancy on a 0–6 scale. The multiple rating scales utilized in the PCSI complicates comparisons across age groups; however, rescaling prevents an inaccurate misinterpretation that younger children have higher stability because the scale range is smaller. Original GMDs values for the PCSI-SR5 and PCSI-SR8 can be found by dividing values reported in this manuscript by three. IN addition, GMD values reported are group averages. Therefore, some individuals may have had large changes (low stability) in their retrospective pre-injury recall. Clinicians should be aware of this when working on the individual patient-level.

Symptom reporting is limited by both floor and ceiling effects, floor effects being a larger concern in our study evaluating pre-injury ratings. The GMD looks at the absolute value of differences in pre-injury reporting over multiple time points. Patients who consistently report no symptoms would have a GMD of zero, thus increasing the stability of the group analysis. However, even patients at the floor (total score of zero) can have a large GMD if they report higher pre-injury ratings at subsequent assessments. Recovery status is not considered in this study. Some participants will have reached full concussion recovery during the follow-up period while other were still experiencing post-concussion symptoms and deficits. How recovery status may have affected retrospective symptom reporting is unknown. Nearly all patients preferred email follow-ups, but a minority of patients (*n* = 276) requested phone follow-ups. Similarly, very young children may need their parent to help read or understand the symptom checklist. It is possible that interacting with a research assistant via phone follow-ups or parent may influence the patient's response.

While some items underperformed relative to other and some minor differences in stability between the full sample and the + Pre-Injury Ratings reporters were apparent at the item-level, the stability for both groups was high at both the subscale and full-scale level. Based on the results of this study, there appears to be no additional clinical benefit to having patients retrospectively recall their pre-injury symptom levels multiple times throughout the recovery period and this can reduce burden on the healthcare provider and the injured child. If multiple retrospective pre-injury ratings are needed (e.g., seeing multiple providers), clinicians can be assured that children and adolescent will retrospectively rate pre-injury levels with relatively high stability. As long as clinical decisions are based on the subscale or the full-scale level, multiple pre-injury ratings will not have meaningful differences and provide equivalent results.

Children and adolescents retrospectively rate pre-injury levels of post-concussion symptoms with good-to-perfect stability over the first 3-months following their injury. Although some individual items underperformed, variability was reduced as items were combined at the subscale and full-scale level. There is limited benefit to multiple pre-injury symptom queries.

## Data Availability

The dataset for this study can be requested through Ontario Brain Institute (OBI) BRAIN-CODE after registering using the following link: https://www.braincode.ca/user/register

## Ethics Statement

This study was carried out in accordance with the recommendations of the Research Ethics Board of each participating institution with written informed consent from all subjects. All subjects gave written informed consent in accordance with the Declaration of Helsinki. The protocol was approved by the Research Ethics Board of each participating institution.

## Author Contributions

ET, RZ, GG, and IG were involved in the conception of this project. KT made substantial contributions to data analysis. ET, KT, MS, and CV were involved in the interpretation of the findings. ET drafted this manuscript and all authors critically revised the manuscript for important intellectual content. All authors approved the final version and agree to be accountable for all aspects of the work.

### Conflict of Interest Statement

The authors declare that the research was conducted in the absence of any commercial or financial relationships that could be construed as a potential conflict of interest. The handling editor declared a past co-authorship with one of the authors IG.
