# Supplementary material for: The Stability of Retrospective Pre-injury Symptom Ratings Following Pediatric Concussion
Source: Front Neurol. 2019 Jun 27;10:672. doi: 10.3389/fneur.2019.00672 (PMC6610489; doi:10.3389/fneur.2019.00672)
Supplement: Supplementary file 1 [file Data_Sheet_1.docx]

Supplementary Material

# Supplementary Figures and Tables

As the Gini’s Mean Difference (GMD) is a statistical outcome many readers may be unfamiliar with, we have created a brief illustration for how the GMD is calculated to aid in the understanding of our results and conclusions. In addition, we have provided a table to more thoroughly report missing data throughout the study.

**Supplementary Table 1**. Mock dataset with 10 participants who completed the PSCI-SR13. The numbers in the ER and 1, 2, 4, 8, and 12-week columns represent patients rating of “headache” throughout their enrollment in the study. The GMD is calculated in the last column. More specific detail on how the GMD is calculated is provided in Supplementary Table 2 and the GMD Equation.

| **Patient** | **ER** | **1-week** | **2-weeks** | **4-weeks** | **8-weeks** | **12-weeks** | **GMD** |
| --- | --- | --- | --- | --- | --- | --- | --- |
| 1 | 0 | 0 | 0 | 0 | 0 | 0 | 0.00 |
| 2 | 0 | 0 | 0 | 1 | 0 | 0 | 0.33 |
| 3 | 0 | 1 | 0 | 1 | 0 | 0 | 0.53 |
| 4 | 0 | 1 | 0 | 1 | 0 | 1 | 0.60 |
| 5 | 0 | 2 | 0 | 0 | 0 | 0 | 0.67 |
| 6 | 0 | 2 | 0 | 0 | 1 | 0 | 0.87 |
| 7 | 0 | 1 | 2 | 0 | 1 | 2 | 1.07 |
| 8 | 0 | 2 |  | 2 | 0 |  | 1.33 |
| 9 | 0 | 2 |  |  | 2 |  | 1.33 |
| 10 | 0 |  |  | 3 |  |  | 2.00 |

| **Comparison** | **Assessments** | **\|Δ\|** |
| --- | --- | --- |
| 1 | ER to 1-week | 2 |
| 2 | ER to 2-weeks | 0 |
| 3 | ER to 4-weeks | 0 |
| 4 | ER to 8-weeks | 1 |
| 5 | ER to 12-weeks | 0 |
| 6 | 1-week to 2-weeks | 2 |
| 7 | 1-week to 4-weeks | 2 |
| 8 | 1-week to 8-weeks | 1 |
| 9 | 1-week to 12-weeks | 2 |
| 10 | 2-weeks to 4-weeks | 0 |
| 11 | 2-weeks to 8-weeks | 1 |
| 12 | 2-weeks to 12-weeks | 0 |
| 13 | 4-weeks to 8-weeks | 1 |
| 14 | 4-weeks to 12-weeks | 0 |
| 15 | 8-weeks to 12-weeks | 1 |

**Supplementary Table 2 and Equation 1**. GMD is calculated by taking the sum of the absolute value of each pairwise comparisons divided by the number of comparisons. Here is an example for Patient 6 from Supplementary Table 1. The absolute value for each pairwise comparison is shown in the Table and the final calculation for the GMD is shown in the Equation.

$$GMD=\frac{Sum\left| \Delta\right|}{Total Comparison}=\frac{2+0+0+1+0+2+2+1+2+0+1+0+1+0+1}{15}=\frac{13}{15}=0.87$$

**Supplementary Table 3**. Presented is the percentage of each age group with missing data for each symptom. Information is available for all time points, where PED= the initial PED visit, FU1= the 1-week follow up, FU2= 2-week follow up, FU4= 4-week follow up, FU8= 8-week follow up, F12= 12-week follow up, GMD Unavailable= GMD could not be calculated (participant only completed one time point; not included in analysis).

| **Item** | **Missing PED (%)** | | | **Missing FU1 (%)** | | | **Missing FU2 (%)** | | | **Missing FU4 (%)** | | | **Missing FU8 (%)** | | | **Missing FU12 (%)** | | | **GMD**  **Unavailable (%)** | | |
| --- | --- | --- | --- | --- | --- | --- | --- | --- | --- | --- | --- | --- | --- | --- | --- | --- | --- | --- | --- | --- | --- |
|  | **5-7** | **8-12** | **13+** | **5-7** | **8-12** | **13+** | **5-7** | **8-12** | **13+** | **5-7** | **8-12** | **13+** | **5-7** | **8-12** | **13+** | **5-7** | **8-12** | **13+** | **5-7** | **8-12** | **13+** |
| Headache | 3.9 | 2.5 | 1.6 | 13.7 | 13.9 | 15.2 | 13.7 | 14.5 | 15.6 | 15.4 | 14.4 | 17.3 | 20.2 | 19.7 | 22.0 | 24.2 | 24.7 | 28.6 | 6.6 | 6.2 | 6.7 |
| Nausea | 4.3 | 2.5 | 1.7 | 13.9 | 14.0 | 15.2 | 13.7 | 14.4 | 15.8 | 15.9 | 14.5 | 17.4 | 20.2 | 20.4 | 22.2 | 24.0 | 24.7 | 28.6 | 6.6 | 6.2 | 6.7 |
| Balance | - | 2.5 | 1.7 | - | 14.0 | 15.2 | - | 14.4 | 16.0 | - | 14.7 | 17.2 | - | 20.1 | 22.1 | - | 24.9 | 28.8 | - | 6.2 | 6.7 |
| Dizziness | 4.1 | 2.6 | 1.7 | 13.9 | 13.9 | 15.4 | 13.7 | 14.7 | 15.9 | 15.7 | 14.7 | 17.2 | 20.0 | 20.2 | 22.5 | 24.2 | 24.8 | 28.7 | 6.6 | 6.2 | 6.7 |
| Fatigue | - | 2.7 | 1.6 | - | 14.0 | 15.2 | - | 14.6 | 15.9 | - | 14.4 | 17.4 | - | 20.1 | 22.2 | - | 24.8 | 28.7 | - | 6.2 | 6.7 |
| Drowsy | - | 2.7 | 1.6 | - | 14.2 | 15.2 | - | 14.7 | 16.0 | - | 14.6 | 17.1 | - | 20.1 | 22.3 | - | 24.9 | 29.2 | - | 6.2 | 6.7 |
| Sensitivity to Light | - | 2.7 | 1.6 | - | 14.1 | 15.1 | - | 14.7 | 15.6 | - | 14.6 | 17.3 | - | 20.1 | 22.4 | - | 24.7 | 28.8 | - | 6.2 | 6.7 |
| Sensitivity to Noise | - | 2.6 | 1.6 | - | 14.0 | 15.2 | - | 14.6 | 16.0 | - | 14.5 | 17.3 | - | 20.1 | 22.2 | - | 24.9 | 28.8 | - | 6.2 | 6.7 |
| Irritability | 4.3 | 2.6 | 1.7 | 14.4 | 14.4 | 15.2 | 13.9 | 14.6 | 15.8 | 15.9 | 14.7 | 17.6 | 20.2 | 19.8 | 22.2 | 24.3 | 24.8 | 28.7 | 6.6 | 6.2 | 6.7 |
| Sad | - | 2.7 | 1.7 | - | 14.0 | 15.6 | - | 14.7 | 16.1 | - | 14.8 | 17.4 | - | 19.9 | 22.2 | - | 24.8 | 29.0 | - | 6.2 | 6.7 |
| Nervous | - | 2.7 | 1.7 | - | 14.2 | 15.2 | - | 14.7 | 15.7 | - | 14.7 | 17.2 | - | 20.0 | 22.5 | - | 24.8 | 29.0 | - | 6.2 | 6.7 |
| Emotional | - | - | 1.7 | - | - | 15.3 | - | - | 16.0 | - | - | 17.3 | - | - | 22.5 | - | - | 28.6 | - | - | 6.7 |
| Move Slowly | - | 2.7 | 2.1 | - | 14.3 | 15.5 | - | 14.5 | 15.9 | - | 14.4 | 17.6 | - | 20.3 | 22.3 | - | 24.8 | 28.9 | - | 6.2 | 6.7 |
| Mental Fog | - | 3.0 | 1.9 | - | 14.5 | 15.5 | - | 14.9 | 16.0 | - | 14.9 | 17.2 | - | 19.9 | 22.1 | - | 24.8 | 28.9 | - | 6.2 | 6.7 |
| Difficulty concentrating | 4.1 | 2.7 | 1.8 | 14.0 | 14.0 | 15.2 | 14.0 | 14.8 | 15.8 | 15.5 | 14.5 | 17.6 | 20.2 | 19.9 | 22.1 | 24.5 | 24.9 | 28.7 | 6.6 | 6.2 | 6.7 |
| Difficulty remembering | - | 2.7 | 1.8 | - | 14.2 | 15.2 | - | 14.7 | 16.0 | - | 14.6 | 17.5 | - | 19.7 | 22.1 | - | 24.8 | 28.9 | - | 6.2 | 6.7 |
| Vision | - | 2.7 | 1.8 | - | 14.4 | 15.2 | - | 15.1 | 16.3 | - | 14.4 | 17.2 | - | 20.2 | 22.2 | - | 24.9 | 28.9 | - | 6.2 | 6.7 |
| Confused | - | - | 1.8 | - | - | 15.2 | - | - | 15.9 | - | - | 17.1 | - | - | 22.2 | - | - | 28.9 | - | - | 6.7 |
| Clumsy | - | - | 1.8 | - | - | 15.4 | - | - | 16.0 | - | - | 17.3 | - | - | 22.1 | - | - | 29.0 | - | - | 6.7 |
| Answer Slowly | - | 2.7 | 1.9 | - | 14.1 | 15.4 | - | 14.6 | 16.0 | - | 14.4 | 17.5 | - | 19.8 | 22.4 | - | 25.0 | 29.0 | - | 6.2 | 6.7 |
